# Chicago Dental Society

**Published:** 1867-09

**Authors:** 


### CHICAGO DENTAL SOCIETY.

### ADDRESS OF PROF. H. E. PEEBLES.

At the regular monthly meeting of this Society, held on the evening of July 1st, a resolution was unanimously passed inviting Prof. H. E. Peebles, of Saint Louis, to deliver an address before the Association, at his earliest convenience. This invitation having been kindly accepted, the members of the Society, together with a large number of the Dental and medical profession of the city, assembled in one of the public halls, on the evening of July 12th, and listened to the address which follows.

The occasion was one of great interest, and will long be remembered by the profession in this vicinity ; and the high standard of Dental education, advocated by the speaker, received unanimous endorsement.

Upon the close of the address the following resolution was offered by Dr. M. S. Dean, and which prevailed unanimously :

*Resolved*, That the thanks of this Society are hereby tendered to Prof. H. E. Peebles, of the Missouri Dental College, for his very able and interesting address delivered before this Association, and that he is hereby requested to furnish a copy of the same for publication.

*Mr. President and Gentlemen Members of the Chicago Dental Society, and Gentlemen Visitors:*

Through the politeness and urbanity of this Society you are assembled together this evening to listen to a very hastily prepared address from one who feels his inadequacy to the task imposed, and begs your kind indulgence to the many faults that must of necessity appear in a paper so hurriedly gotton up.

The Chicago Dental Society was organized in January, 1864, with about a dozen active members, to which were added eleven honorary members, selected from the Dentists of eight different states. Taking three of that number from the state of Missouri, and city of St. Louis, in which number your speaker has the honor to be counted, and for this honorable distinction placed upon the Mound City, I tender the Society our grateful acknowledgments.

The late venerable Dr. E. W. Hadley, was the first President of your Society. I had the pleasure of meeting Dr. Hadley only once. Then he made a very favorable impression upon me. Dr. H. finished his work and closed his office in 1865, and went to *his* Father and *our* Father to receive his reward. How sad you all felt while standing around his grave on that memorable 6th day of March, to see the remains of your presiding officer deposited in the tomb. But such is our common doom, and oh, how dark would be that tomb if the blessed Jesus had not lain there, and arising opened up the living way to a better world.

During the two years and a half of your existence as a Society, you have met, no doubt, with some discouragements, and some opposition, as every association of men for good purposes does meet. But from your records I see that you have gone on in the path of duty, and greatly increased in numerical strength, even doubling your membership. And by your regular monthly meetings and discussions, you have grown in station and understanding as practitioners, to say nothing about the increase of social feeling and professional courtesy. The Chicago Dental Society is a tower of strength. This Society is shedding a light upon the minds not only of its members, but upon the mind of every Dentist in this great city, from the highest to the lowest. Some may deny this, others may be too low in the scale of enlightenment and progress yet to perceive this fact. To you belong the honor and responsibility of the organization of a State Society in Illinois.

Go on brothers, and do your duty, and leave results to God and posterity.

Gentlemen:—In presenting the subject I have chosen for this address, EDUCATION, I have had to draw somewhat from the thoughts of other men.

The time that has elapsed since I received your very flattering invitation, and the present, and I have had so many duties to perform in the meantime, that I have been unable to do more than make this rough draught in pencil. A decent copy, much less a condensation of the matter were wholly out of the question.

With these preliminary remarks, I shall proceed to the discussion of the subject.

Dentistry is a specialty of medicine. A specialty cannot be separated from the parent stock, and be maintained as an independent profession.

Dental Surgery depends upon the same general principles, and is governed by the same laws that govern general surgery. Hence, the same course of study, and the same general teaching, that is demanded in the one case, is necessary in the other, as a foundation upon which to predicate the specialty. Therefore, if the Surgeon is a Physician—a Doctor—so is the Dentist, the Aurist, the Oculist, for all are specialists in medicine.

No specialty can with impunity do that which will bring odium upon medicine. The special practitioner, is as much and as strongly bound by the laws of honor, etiquette, and courtesy as the general practitioner is.

With these propositions before us, we will proceed to enquire into the present standing and condition of our specialty, in our own country; also, casting a retrospective glance back over a few decades of years, we will compare Dentistry *then*, with Dentistry now, and undertake to show an unprecedented advancement in our specialty over any other department of medicine. We will also enquire into the cause of this progress and show its results, in securing for us a place and a standing in the great family of medical men. In this investigation, it may be argued, that the main sources of our success—our progress up to the present time—are now well nigh exhausted, and that it behooves us to look for other and more enduring means of growth and progress as a specialty, or the time will come, and is now near at hand, that we may be outstripped and fall in the rear.

A thorough and correct medical education, preceded by, and resting upon a good academic course of mental discipline and literary acquirement, must henceforward be regarded as the basis of our superstructure.

The organic elevating machinery of our specialty consists mainly at the present time, in association, journalism, and the lecture room.

There are now over forty Dental societies, in good working order in the United States of America; and a year scarcely passes in which one more such association is not organized and added to the list.

There are now, I believe six Dental periodicals published; the seventh is to commence in October next, and an eighth is in contemplation and *may* issue very soon.

We have also six Dental colleges in full operation now, and our New England brothers are preparing to open the seventh, in Boston, upon the same plan and basis as the MISSOURI DENTAL COLLEGE.

And we trust the time is not far in the future, when the necessity will become apparent to the *profession*, that our specialty must needs be taught by regular curriculum in the great City of the Lakes. And here too, like in St. Louis, she will open her halls, her lectures, museums and hospitals to her young and beautiful daughter, and bid her enter and share in the feast of medical science.

PROGRESS is stamped upon our escutcheon. Onward and upward are our watchwords. We have not run long, but we have run well. The Goal is not yet in sight. The way is long and weary. Shall we tire and faint by the way? Shall we grow weary and slacken our pace?

Nay, but let us like the true knight, face and fight all enemies, brave manfully all difficulties, and put forth strength and energy commensurate with the demand and emergency, and victory will finally perch upon our standard; and the laurel wreath will encircle the brow of the conquerer. Though the present hearers and speaker will be laid low beneath the cold clods of the valley, and our sons, and their grand sons may have come and gone, before the halcyon days arrive; still the time *will* come, when Dental Surgery will stand *pre-eminent* as a specialty in medicine.

See, my brothers, where we stand to-day! Does the learned physician, or the eminent surgeon, fear to cordially greet you, or lock arms with you in the street? Are we not treated as equals—as brothers, and why is this so? We are still but in our infancy as special practitioners. For when your speaker commenced practice in 1836, there was no Dental society—no Dental periodical, or Dental college in the world, nor had there ever been any such thing heard of. Thirty years have not yet elapsed since the organization of a little society in New York, from which sprang the American Society of Dental Surgeons. This society soon felt the necessity for a journal to publish their proceedings and transactions, hence, the *American Journal of Dental Science.* The cultivation and exercise of the talent found in this association developed the necessity for a school to teach the peculiar art and elucidate the science of Dentistry, and hence in 1839, the General Assembly of the State of Maryland was induced to grant a charter for a Dental College, in the city of Baltimore.

The American Society of Dental Surgeons embraced in her membership, the very best talent there was in the Dental ranks at the time. And to her honor be it spoken she did much good, and performed many noble and generous acts. But alas! she committed the egregious blunder of indulging in transcendental legislation, and prescribed the *kind of materials* that she considered ROYAL, and which might be used by her subjects, and they continue to be *loyal.* She, like governments of greater magnitude struck upon the hidden rock of reserved rights, and soon became a thing of the past.

The Mississippi Valley Association soon followed in organization, and now stands as the senior sister of the Western Dental Society; the Indiana State Dental Society; the American Dental Convention; the American Dental Association, and a host of state, district and city societies.

In the meantime, the Baltimore College of Dental Surgery, the Ohio College of Dental Surgery, the Pennsylvania College of Dental Surgery, the Philadelphia Dental College, the

New York College of Dentistry, and last, but not least the school your speaker has the honor to represent here this evening, the MISSOURI DENTAL COLLEGE, were successively gotten into operation; and each one has shown its fruits, and all are doing good service in building up the little mole hill of Dental science and art.

To our specialty belongs the honor of the introduction and application of one of the greatest achievements of modern science. That which has relieved more human suffering and pain than any one thing, in all the wide range of medical learning. I mean anæsthetics. And to us belong all, or nearly all the improvements made in the manufacture and administration of chloroform and nitrous oxyd.

Some of the nicest histological examinations and elucidations have been made under the microscope of the Dentist. And what surgeon has ever performed a nicer operation than that of neurosection of the Dental pulp; by which its normal condition is maintained, and a covering of secondary dentine induced.

And gentlemen, we all, even the youngest of you, who have been regular attendants upon the meetings of Dental societies, know that in our discussions we manifest a healthy and vigorous growth in scientific attainment. Just look back a few years and you will see that "professional fees," "hard rubber," "best materials for filling," etc., engrossed the entire attention of the gentlemen in session! But how stands the case now? "Histology," "Physiology," "Pathology" and "Education" come forth as the prominent subjects for discussion. In each of the two State society meetings, this summer, west of the Mississippi river, there were four elegant microscopes employed to ellucidate Histology, Physiology, and Pathology. These facts need no comment. They are patent to all.

Each and all these things, gentlemen, speak for us, and give the reasons why the physician and surgeon cordially grasp your hand as a respected and beloved brother and fellow in the great family of doctors.

Yes, it is because we have been up and doing—we have been learning—we have been trying to educate and to elevate ourselves, and to grow respectable. For, in professions and callings as in individuals, when they become respectable, they *will be* respected.

Although we began far down in the social and professional scale, we began no lower than the surgeon or the physician. And certainly we have made as rapid progress in knowledge, science, elevation and *education*, as any of our sister specialties, or even our venerated mother, general medicine.

Then, if these propositions be true, as we assume they are, it becomes us to enquire into the best modes of education, and how we shall proceed in the future so as to maintain the ground we now hold, and to advance in as *safe*, if not as rapid marches up the steep and rugged hill of science, and across the thorny plains of human wisdom and intellection.

The indomitable energy of the honest, earnest workers in the Dental ranks, who, seeing the true position of things, and understanding at a glance, that to elevate ourselves as specialists we must work, we must study, we must learn, have laid their shoulders to the wheel, and the car has moved on steadily.

The great fascination of the nicety of manipulation, has drawn out the best efforts and the highest skill of the Dental operator. The laudable desire to imitate the Great Creator, and repair the damages wrought by decay upon the pearly arches of beauty, in His noblest work; as well as the encouragement given by the liberal minded, and noble hearted physician, have all contributed to inspire the Dentist with an ardent love for his calling. These several considerations have aided us in our rapid advancement. But having thus attained unto a certain point of proficiency other aids and stimuli must be invoked, or we must fall in the wake.

We are far advanced in the art, but can the art precede the science? "Can practical application go in advance of discovery of data? Can implements be made without material?" "Can fabrics be woven without both implements and material?" Useful arts can therefore make no more rapid development than science. "In fact, art is usually behind science by long stretches of distance, and these sometimes so great that the popular voice, not discovering their mutual dependence, sometimes clamors against science as dreamy and unworthy, because it seems useless." "Science is its own justification; it needs no defence any more than *truth*; the one is synonymous with the other."

"But the mass of mankind are most concerned to secure the comforts and benefits of useful arts. The needs of the body, the happiness of society must be provided for."

"Men have a right to demand of science, that she do not shut up her treasures as ore in the bowels of the earth, but freely yield them up to be fashioned into implements, for useful labor and to minister to the happiness and welfare of the race." Humanity and benevolence enter with authority to compel the assent of science to their mandates.

There frequently occurs in noble minds a sore conflict between the demands of pressing labor and the monitions of conscience and of the sense of duty. As such a one, to the best of his skill, administers to the distresses of men, he fears that his mind may not possess all the facts, which late investigation has afforded upon this or that case; or that his hand may not wield the instrument with the correctness and skill which other men have acquired. What operator of twenty years practice has not felt this burden? What young Dentist in his first years has not been harrassed by the fear that he is not doing justice to his patient, because he may not be fully competent in the knowledge of the case?

"To what purpose is our Dental journalism but to furnish to the anxious, waiting practitioner the latest facts, discoveries and modes of operation or treatment; to be at once seized upon for the good of our patients?" Why then fill their valued pages with the mere details of the business matters transacted in local societies?

But is any Dental practitioner bold enough to say, that he has explored the records of the past—has stored his mind with all the wisdom of recent years, and is every year up to the level of modern science, so that he comprehends within his grasp all of science, and can apply his art with the highest human skill? "That no case can be presented to him in which he cannot offer the best and soundest advice possible, among the Dental profession. Can settle a diagnosis with a precision which no other can excel. Can perform an operation with a dexterity inferior to no other?"

"Such perfection of skill and knowledge, no man of ordinary sense, modesty or veracity will venture to affirm of himself. The assumption would to his fellows be the strongest evidence of his imperfections."

"As the decades pass, and science and art attain greater completeness, the labor of the Dentist in fitting himself for his profession, becomes more and more heavy. As life advances, and the cares of increasing practice multiply, his disposable time for study, of both, his own and other men's labors becomes less and less, until it may be reduced to the merest fraction of a day."

Still, perhaps, no class of men strive harder than medical men do, to keep fully up to the times, in the sciences more nearly and directly effecting the art they practice; but too often they are the first to accuse themselves of being unable to meet the duties of their daily calling, and keep pace with modern improvement. And it will be observed that the men ready to make this confession are, or have been, the most studious, the best qualified practitioners among us.

Many a man, a willing votary of science, deplores the fact, that he is so swallowed up in the cares, the labors and the hurry of practice that neither energy or time remain for the quiet pursuits, to which he would gladly turn. "Could the human frame endure heavy encroachments on the hours of sleep, and this for long periods, or a long life, what rich contributions would multitudes of general practitioners and Dental surgeons bring to their respective sciences. This unhappily is impossible, as many an overwrought brain discovers; but seldom in time to secure its longer services in the cause of science and human improvement.

The arduous physical and mental labors of the truly ambitious and industrious operator and student in our ranks, too frequently urged with an imprudent zeal, are often stopped by death. "The man fitted mentally to do most and best, feels keenly the brevity of earthly career, and strives by diligence too great for human endurance, to make life most fruitful. But alas, how often is he and his friends, disappointed in the promise of a rich harvest, because the Great Reaper thrust the sickle into the laborer's field."

"All medical men agree in seeking the growth and improvement of their art. All admit that its science should steadily and unfalteringly move forward. All will admit that the art cannot in any large sense move onward faster than the science, and that the true way to better the art is to enlarge the science."

The air, the earth, the sea, must contribute to soften the hardships of social condition, to cure the sickness of men; to soothe the pains of decay and dissolution.

"This brings us to the subject of medicine. It is both a science and an art. As a science, far in advance of its early beginnings, yet mayhap as far yet from its ultimate perfection as from its primeval state. As an art its efficiency and success depend upon the fullness and clearness with which its facts are learned and logically systematized; upon the memory and readiness of the practitioner; upon the skill of his manipulations; upon the fertility of his inventive and adaptive power; and upon the keenness of his senses, touch, sight, hearing, &c. To successfully practice an art, demands first the careful study of science and also the education of the individual; or, as I may express it, taking the practitioner in the sense of an instrument, he must be fashioned into shape and fitness in both mental and physical qualities, before he can deal with the facts of science in their application. To make a fabric for wearing apparel, science must discover the crude cotton, silk, or wool, and prepare it for use; while art must invent the weaving machine and bring it to perfection. The physician possesses himself of facts furnished by science, which are his raw material, and then proceeds to qualify himself to apply them to his healing art.

In the economic arts it may matter little if there be a wide discrepancy between the advanced state of science and the clumsy appliances which utilize it. Convenience and luxury may be lacking, but perhaps nothing more. In the art of medicine there must be no such discrepancy. Human woe and bodily privation, the loss of health, the loss of the senses, and the loss of life are the subject matter of this art. Here there may be no lagging behind the front line of scientific attainment; the very fore front is where the disciple of the *ars medendi* must place himself and remain. He has no business in the rear; he is recreant to himself, recreant to humanity, recreant to duty and to religion, if he voluntarily stay behind in the onward march of medical science."

"It is not to be denied that some men obtain extraordinary success in grasping a multitude of facts, and in reducing them to systematic and logical order. They are the master minds of their age, and their names will be enduring as human memory. But gifted men, like Erasmus, Bacon and Humboldt, are the astonishment of mankind, and were we obliged to wait for their advent in the cause of science, the accumulation of knowledge would be at the rate of progression in geological eras, while the mode of progress, instead of being by gradual accretion, would be an alternation of long and dreary ages of stagnation with brief times of dazzling splendor. Such, however, is not the order of things. The processes of nature and the growth of human knowledge are alike.

Increase is by slow additions, by patient and pains taking observation. A multitude of workers, each bearing the little burden which he has gathered from the path where he has wandered, cast in their contributions, and as the decades pass the stately pile of human knowledge creeps upwards; it slowly assumes harmonious proportion, broadens its base, and lifts its soaring height. The rising of an ant hill, the building of Cheops, in one sense are typical of the increase of science; but these limited and finite models are far from being perfect representatives, for science is the gleaning of gems from the exhaustless mines of knowledge, whose storehouses are the recesses of the infinite mind; to neither may we venture to set bounds."

"In actual fact, regarding the practitioners of medicine, surgery or Dentistry as we find them, does not every one know that but very few of them possess the highest fitness which medical knowledge and skill can reach? This is largely due to defects of education, both of an academic kind and in the schools of medicine and Dentistry. Men are often compelled, by the need of getting a livelihood, to enter practice, feeling themselves yet very inadequately prepared.

"Want of fitness at the outset of medical life must to a greater or less degree be affirmed of every one. Youth cannot claim the attributes of age and experience. If in this regard the young doctor is at a disadvantage, neither on the other hand ought the standard of medical attainment to be purely theoretical and transcendental. It must be such as the wisdom of experienced and practical men deems needful. It must fairly represent the present; it were folly in imposing medical qualifications to seek to discount the future."

It may then be asked, can any specialty in medicine be fully acquired, or thoroughly taught in a school, when the general principles of medical science are not taught in the fullest sense of the term?

Can the anatomist, thoroughly instruct his class in "ocular anatomy," or "aurical anatomy," or "dental anatomy," to the exclusion of general anatomy? Can the engineer teach boiler engineering to one; cylinder engineering to a second; piston engineering to a third; cam engineering to a fourth; and so on to the end of the various parts of his complex machine; yet, far simpler than the machinery of the human frame that is "wonderfully and fearfully made?"

Or, can the physiologist teach a special physiology, and show us the functions of a set of organs necessary to sight, or to touch, or to hearing, &c., without teaching the general range of his chain? Or can the pathologist, demonstrate to his class, how and why disease exists in the eye, the ear, or the mouth and teeth, and not examine the condition of other organs and their functions? Does not the intimate and complete connection of the sound parts with all their nice relation to, and dependencies of each one upon the whole; as well as the general circulation of the blood; the distribution and all pervasion of the nervous system; the common centers of alimentation and respiration, all tend to prove the absurdity of exclusive special teaching?

The man who claims to be a well qualified Dentist, and gives no heed to general medicine in his preparation, or he who claims pre-eminence upon his mechanical skill alone, is as little of a *true Dentist* as the stone cutter, who chisels the ashlar to the requirements of his rule and square is an architect, compared with the man of talent and science who plans and rears the noble edifice.

"Medical science differs from nearly all other sciences." Chemistry pursues the labors of the scales and the retort. Botany seeks light and knowledge in the field and the forest. While geology is busily hammering the rocks, or boring deep down into the bowels of mother earth. But medical science has to deal with a peculiar organism. "The subject or patient if you please is a man; his whole organism is before us; it may be only partially diseased, but that part bears intimate relations to all other parts; and sometimes is inseparable from the whole. No part or organ can be isolated from the rest of the body in health ; nor can it be isolated in the phenomena of disease."

The specialist, be he oculist, aurist or Dentist, has no just claims to the honors of a medical gentleman, who has not striven to gain a thorough knowledge of general medicine.

The science of medicine, in its general range, is not taught in our schools of specialty, as they are generally constituted. Nor will it be, so long as the absurd notion of making a *mere* specialist, is kept up, and our schools ignore the fact that a thorough knowledge of general anatomy, physiology, pathology and therapeutics is essential to the education of our specialists.

Where is the author of "aural physiology?" Who has given us an "ocular pathology?" And yet would either be more remarkable than "Dental chemistry," or chairs of "Dental physiology," or "Dental pathology?" Such marks of weakness, faulty judgment, and inexperience are incident to our youthful days as a specialty, better things may be expected of us ere long. Our schools will necessarily enlarge their curriculum, and widen their range of instruction. This, I think, is clearly indicated by recent developments.

If the true physician seeks to enlarge his knowledge beyond the immediate confines of his profession. "The specialist can lay no claim to honor and respect, if he permit himself to shrivel into the scanty limits of his little shell, and know nothing outside of its impenetrable crust."

He should be well educated in general medicine and surgery, and in all their departments. He ought to know them practically. If practicable, he should seek this practical knowledge in general hospitals.

When thus made ready for the wide responsibility of general practice—then let him turn his attention to his selected specialty, and select that school where the widest scope is given, and the most ample curriculum is presented.

"Let the specialist take this ground, that he has mastered all the preliminary studies which every physician pursues when he sets forth in his career, and then let him add to this preparation the further labors of his chosen department, cultivated with ardor, and to a degree which puts him in this particular qualification visibly above the attainments of his fellows, and he then need not fear a want of recognition and respect. His fellows in the profession must and will respect him. He is one of them; he never secedes from their ranks, nor will they have the least disposition to cast him out."

"He is jealous of professional honor—he is mindful of professional courtesy. He is none the less bound by ethical rules than are his brethren. His real position toward them is that of a *counsellor in difficult cases belonging to his sphere.* He claims a peculiar skill on one subject; when other practitioners need counsel in these cases, they ask for his assistance. They may simply call him in consultation, or they may turn the patient over to his care. In either case the specialist must govern himself by the rules which all medical men observe in holding consultations with each other."

"Specialists have sometimes demanded, as their right, that they may advertise their pretensions in the public prints or in the medical journals. The former kind of self-proclamation is universally condemned by the profession, as an unworthy attitude for the member of a liberal profession to hold towards the community; none the less should it be condemned and abnegated by the specialist. Advertising in *medical journals* may be done as offensively as in any other prints."

But prior to all this, professional education then must be a solid foundation laid, a broad basal structure erected, in the mind of the youth; by a thorough course of discipline and instruction upon which to build the beautiful edifice of professional learning and usefulness contemplated in the foregoing remarks.

I have just read a paper on education in one of our most popular weeklies that seems to cover the ground now contemplated in the organization of a system of intermediate schools of such a character that a youth could there be fitted for any of the ordinary pursuits of life. And also, the establishment of one grand university in the West, where all science would be taught, and from whence may emanate scholars, statesmen, lawyers, physicians. surgeons, dentists, etc. And I now beg your indulgence while I read an extract or two from this valuable paper:

"We are aware that in the present condition of education and the material upon which it has to act, above all with the pressure upon young men, which is on the increase, to complete their educational course at the earliest possible date, the number of those likely to enjoy the inestimable moral and mental advantages in the discipline of the faculties which the Latin and Greek, more than any other languages afford, can not, as compared even with the better class of the population, be great. We do not expect to see the standard of Oxford, or Cambridge, or Salamanca attained, save in individual instances; but we do contend that our higher education should be real and solid as far as it goes, and thorough and complete when it is attempted to be carried out. This must be taken as the illuminating idea of these remarks, and quicken them with their true interpretation.

On no subject, probably, are there more false theories, more vague and erroneous notions, than education ; and this among other causes, from the fact that whilst on the one hand its importance and the interest it excites force it upon the attention of every intelligent mind, on the other, it can not be fairly grasped, nor its principles understood save by those who have, in a measure at least, participated in its substantial advantages. Again, education is the formation of the intellect; but the intellect being a faculty, or the union of several faculties, of the soul, is distinguishable but not separable from it. In forming the mind you necessarily affect the soul. Thus, at the very start, a vast field is opened and most momentous moral questions laid bare.

To form and train the mind is a science and an art. The process, from a Christian standpoint, is based upon one central idea, depends upon certain principles, is carried out by certain methods whose advantage experience has demonstrated; and all this is as much beyond the depth of him who has not mastered the subject as is any other science reduced to an art—the practice of medicine for instance. Now there are two ways in which a mastery of the subject may be gained: either as a result of having been well trained one's self, or through a special and careful study of the question; and even this latter presupposes the groundwork of a liberal education. The number possessing either advantage is more limited than one might suppose. But as so many, now-a-days, undertake to discuss the subject in some or all of its branches or phases, the result is that crudities, absurdities, mischievous theories and pernicious errors are constantly being put before the public in newspapers and other publications, and thus influence the community, and even confuse, blind and lead astray the judgement of numbers otherwise disposed to be docile and right-minded. But the mischief rests not here. These things becoming diffused by frequent repetition, carry with them in that very fact a sort of proof which warrants their being taken for granted; they approve themselves to the mind, and engender kindred ideas and views. Parents endeavor to realize them in the education of their children, and officious public opinion intervenes to claim that they be accepted. Thus is created a demand for certain species or fashions of education; and the demand produces a supply. Some men are found but too willing to cater to these wants, and others from an overruling necessity are forced to imitate, to some extent, their example. Hence "commercial colleges" with their pretence, academies with their sham, colleges with degrees, schools burlesquing universities, universities with A B C pupils, graduates who are only fit to enter the *curriculum*, and the general shallowness of what is called education. (The rage is to learn in the shortest time, and with the least labor. But nothing real and solid is acquired. A little of everything is learned; at bottom nothing.)

Still all this most astonishing unreality with many of its mistakes and errors patent on its face, is termed and by very many supposed to be education. And what is more, it is not only a sober fact which stares every one in the face who investigates the question of liberal culture with a view to aid its advancement in the society in which he lives, but one of the terrible obstacles to be overcome.

Our interests in this respect, it would seem, require first, the establishment of a class of schools whose end shall be clearly understood and well defined, intermediary in their character and limited in their object, and provided with means to enable them to surmount the difficulties growing out of inadequate support, and to ensure a durable existence liable to no flux or decay from individual caprice or the accidents of life, so that they may attain, as near as may be, the end for which they are established. We need a class of schools which will provide for our youth what Eaton, and Harrow, and Rugby, and the German Gymnasia provide for the English and German—a class of schools in which they may be judiciously and thoroughly trained within the limit of the studies proper to such institutions, and sent forth to enter the *curriculum* of the university, or to begin the world if their schooling is to end with their fifteenth or sixteenth year; and *next*, a university—one *real* University in the West.

Education is not the juxtaposition of disjointed, disconnected or incongruous parts, but a homogeneous whole. It starts from a central point, proceeds on certain well settled principles, and advances by rule. These schools, or gymnasia, or colleges, are part of the system. They take up the work where rudimentary instruction ends, and carry it forward to the point where liberal culture properly begins, and no further. They make no preteuce of doing what they are not intended to do, and cannot do. Their aim is to train thorough, not superficial students; to turn out youths, who cannot pretend to many things, but who will really know what they assume to have learned, who have advanced step by step mastering and assimilating their knowledge as they progressed. Nor does any part of this training unfit the youth for any career in the world. None of the studies in this system in case the pupil does not wish to go beyond the school, is lost. For whatever may be said about classical studies by the conceited or the ignorant, it is certain that properly pursued, and so far as pursued they are a positive and an immense advantage, of real, tangible, positive use in after life. As the ink dripping from the pen stains the paper, leaving upon it indelibly the impress of the writer's thoughts, so do these studies impress upon the mind characteristics peculiar to themselves, and tend to develope desirable qualities beyond what any other studies pursued to the same extent can or will.

The classics, mathematics, the vernacular, some foreign tongue, and a science, chemistry or physics, in its elements, comprises about all that is taught; and it needs no venture to affirm, since experience is at hand to prove it, that a youth at twenty, whether he has gone through a full course or not, educated under this system, will compare advantageously, other things being equal, with one of the same age trained under any other.

A great mistake of our day, and the error is deep and widespread, is the notion that there are no fixed, no long-settled principles of education, but that the mind of the youth may be equally well trained under differing and clashing systems —systems whose starting points are the unqualified negation one of the other. If education be, as it is, the disciplining and the formation of the mind, it is evident, since in the average minds are alike, that what is suited to one must be suited to another. What every one must desire is a trained and formed intellect; now the acquirements and the qualities which combine to produce this intellect, are precisely such as are desirable in any walk of life; and the question is simply what course of studies best serves to promote this end. Here is the beginning and immediate end of education; and to attempt to introduce into it arts, and trades, and business pursuits, or "commercial education," as it is called, or practical education, as it is termed, is simply an absurdity. It would be just as sensible to attempt carpenter education, and shoemaker education, and so on to the end of the list of the thousand pursuits of men. We do not say that "commercial education" so termed, so far as it goes and so far as it is education at all, is not good; but for the most part what goes under this name is a pretence and a sham, one of the unrealities of the day.

The drift of these remarks does not imply that our youths are to be trained only to make *scholars* of them when grown men. On the contrary, we have distinctly said that liberal culture is the lot of the few, and we are just now speaking for the many. But we have been contending that it is the interest of the West, to build up a class of schools, which meet a want, which are part of a system, which render a double service by instructing solidly, really educating, as far as they go, those who for one or other reason, do not desire to pursue their studies into the higher branches of learning, and at the same time putting forward those who intend to complete their course—a class of schools without which a university is an impossibility, and liberal culture unattainable.

Nor are we condemning such schools as do the work of "commercial colleges;" aside from their quackery and sham they may serve a useful purpose. But they stand apart, by themselves; they form no part of any system of education. They may be regarded in one respect as sops to Cerberus; they bend to the notions and flatter the prejudices of such as are willing to accept the shadow for the reality.

There is a very large class of boys who are destined by their parents to receive simply what is called in England a *good English education*, with the addition of French or German; and who, without any pretense to scholarship may, and often do, become well informed, well read, and as the word goes, well educated gentlemen. The schools, colleges or gymnasia, be their name as it may, of which we have just been speaking, will by a course parallel to that pursued by those who are aiming at the highest education, serve this purpose. So far as mathematics, the study of any science, a foreign tongue, and the incidental studies of history and geography are concerned, the course should be identical."

In conclusion permit me to say, that while many good and faithful, earnest workers in our ranks have had very few early advantages, like your speaker, and some of these men have by dint of hard work and hard study gained respectable distinction among their fellows, the great majority, have remained in ignorance and obscurity. If we had all enjoyed the opportunities that are *now* afforded to our young men, we might have been much more useful, to our patrons, and creditable to our profession.

The Dentist should be a good, honest, faithful, polite, intelligent, courteous man. In a word, he should be a christian gentleman.

And finally my brothers, if I have succeeded in these remarks, in inspiring you with more zeal in the cause of Dental education, or in causing any, even one single young man to determine that he will lay hold of the opportunities now afforded and make a first class Dentist of himself, by a thorough preparation and a faithful performance of his duties as a Dentist, then I have not spoken in vain; and you have not given me such patient and respectful attention for naught.

